# Supplementary material for: Intrinsic conformational equilibria position arrestin‐2 for activation
Source: Protein Sci. 2026 May 15;35(6):e70627. doi: 10.1002/pro.70627 (PMC13178158; doi:10.1002/pro.70627)
Supplement: Supplementary file 1 — FIGURE S1. 13C‐1D traces from ZZ‐exchange spectra. Representative traces along the carbon dimension for each major and minor peak of I241 and I317 at four representative delay values (10, 100, 250, and 400 ms). All spectra are shown at the same intensity scale. [file PRO-35-e70627-s001.docx]

**Intrinsic conformational equilibria position arrestin-2 for activation**

Tucker J. Shriver^1^, Kerem Kahraman^1^, Mingzhe Pan^2^, Çağdaş Dağ^3,4^, Marco Tonelli^5^, Scott A. Robson^1^, Joshua J. Ziarek^1*^

^1^Department of Pharmacology, Northwestern University Feinberg School of Medicine, Chicago, IL, USA

^2^Department of Cellular and Integrative Physiology, University of Texas Health San Antonio, San Antonio TX, USA

^3^Nanofabrication and Nanocharacterization Center for Scientific and Technological Advance Research (n^2^STAR), Koç University, Istanbul, Turkiye

^4^Koç University Isbank Center for Infectious Diseases (KUISCID), Koç University, Istanbul, Türkiye

^5^National Magnetics Resonance Facility at Madison, Department of Biochemistry, University of Wisconsin-Madison, Madison, WI, USA

^*^Corresponding author: Joshua.ziarek@northwestern.edu

**KEYWORDS:** beta-arrestin, conformational equilibria, pre-activation, interdomain rotation, dynamics, thermodynamics of activation, GPCR signaling adaptor


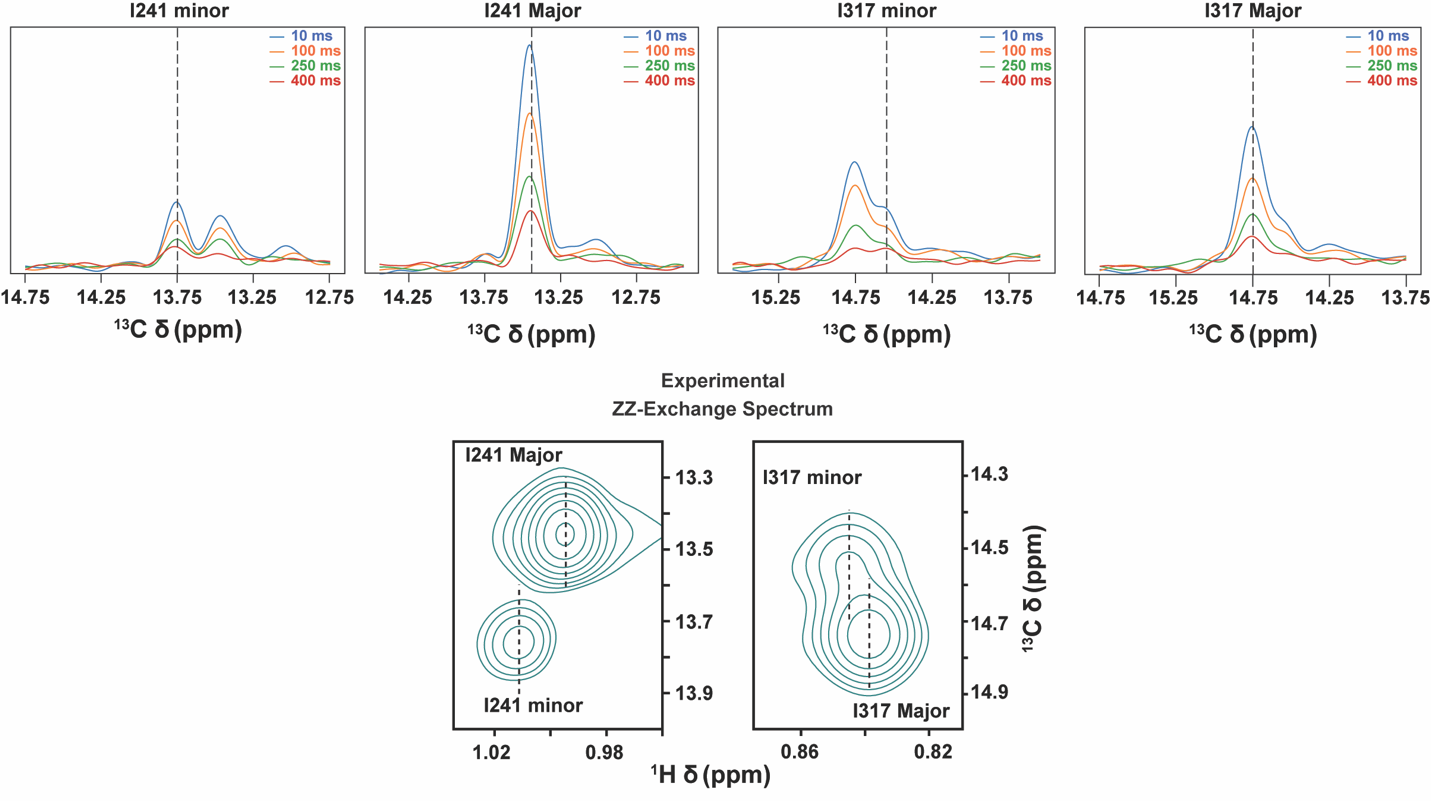


**Figure S1. ^13^C-1D traces from ZZ-exchange spectra.** Representative traces along the carbon dimension for each major and minor peak of I241 and I317 at four representative delay values (10, 100, 250, and 400 ms). All spectra are shown at the same intensity scale.
